# Braithwaite’s Retrospect of Practical Medicine and Surgery

**Published:** 1865-01

**Authors:** 


Braithwaite's Retrospect of Practical Medicine and Surgery. Part I. January, 1865. Uniform American Edition. New York: W. A. Townsend, 55 Walker Street.

This well-known semi-annual publication is still promptly issued; and the contents of the present number are as varied and valuable as those of any that have preceded it.

